# Editorial: Crossing sensory boundaries: multisensory perception through the lens of audition

**DOI:** 10.3389/fpsyg.2026.1848850

**Published:** 2026-05-05

**Authors:** Asterios Zacharakis, Charalampos Saitis, Zachary Wallmark, Argiro Vatakis

**Affiliations:** 1 School of Music Studies, Aristotle University of Thessaloniki, Thessaloniki, Greece; 2 School of Electronic Engineering and Computer Science, Queen Mary University of London, London, United Kingdom; 3 School of Music and Dance, University of Oregon, Eugene, OR, United States; 4 Department of Psychology, Panteion Panepistemio Koinonikon kai Politikon Epistemon, Athens, Greece

**Keywords:** audiovisual interaction, audition, crossmodal correspondences, multisensory perception, perceptual decision-making

Research interest in crossmodal correspondences and multisensory perception has grown significantly over the past 15 years (e.g., Chen et al., 2024; Sathian and Ramachandran, 2019), revealing a network of connections that link sensory qualities across modalities. The 13 papers (co-authored by 40 authors) collected in this Research Topic place sound and audition at the center of this network, spanning modality pairs, methods, and theoretical traditions. Across this diversity, a central distinction emerges between approaches addressing applied multisensory integration and those uncovering novel crossmodal correspondences. Reflecting this divide, most contributions (nine out of thirteen) treat sound as a carrier of adaptive information influencing multisensory task performance, whereas a smaller group (the remaining four) reports either novel audio-centric correspondences or new insights into existing ones, thereby expanding our understanding of how sound relates systematically to other sensory domains.

A closer examination of the multisensorry integration contributions reveals one additional shared characteristic: a focus on audiovisual interaction, arguably the most common sensory pairing in the natural environment. Here, sound co-occurs with visual input in ways that measurably alter perception and decision-making, according to principles of congruence and causal inference, while the listener is conceived as a system optimizing perceptual accuracy under uncertainty.

In this line, Brožová et al. employed a modified implicit association paradigm in which participants performed speeded categorizations of sequentially presented unisensory stimuli, using shared response keys that either align or conflict across auditory and visual dimensions (i.e., pitch with elevation or size), thereby probing crossmodal correspondences. By combining behavioral and neurophysiological data, they showed that congruent response mappings increase the rate of evidence accumulation while leaving the decision threshold unchanged. Meanwhile, Li et al. examined crossmodal interference in both directions, auditory distractors on visual tasks and vice versa. Again, using a combination of behavioral and neurophysiological data, they showed that auditory and visual categorical decision-making are not equally vulnerable to cross-modal intrusion, but instead depend critically on the specific modality pairing. More specifically, auditory distractors interfered primarily with sensory processing, whereas visual distractors modulated post-sensory decision stages. Staying within the audiovisual realm and the behavioral–neurophysiological approach, Morucci et al. employed an auditory cue–image matching task while recording EEG signals and reaction times. Behaviorally, they found faster reaction times when visual object recognition was guided by word cues compared with natural sound cues. Neurally, they observed modulation of alpha and beta rhythms, consistent with the idea that language cues shape anticipatory visual processing toward the expected object.

Three contributions investigated audiovisual interactions in speech and face processing, examining how auditory and visual signals influence each other under different conditions. Magnotti et al. tested Japanese speakers on the McGurk illusion and showed that variability in perception is better explained by probabilistic encoding of audiovisual disparity than by cultural experience. Yu et al. compared real and deep neural network synthetic faces in speech-in-noise tasks, demonstrating that real faces produced the strongest audiovisual integration, while synthetic faces still enhanced integration relative to baseline, but to a lesser extent. In particular, synthetic faces were less effective for fricatives such as /f/, /v/, and /th/, which involve visible interactions between the teeth and lips.

Fremerey et al. recreated the complexity of real communicative environments by testing participants ability to match speech content to visual source in a virtual classroom scenario with up to 10 simultaneous speakers. By systematically manipulating visual realism (360 ° video vs. computer generated imagery) and auditory presentation (binaural vs. diotic), they showed that performance improved with more naturalistic cues, demonstrating both the sensitivity of their paradigm and its potential for studying cognitive audiovisual scene analysis in ecologically valid settings. Exploring the virtual classroom setting from a different angle, Breuer et al. looked into visual interference to an auditory classification task. Expectedly, concurrent visual interference slowed responses, while congruent pairings facilitated performance and incongruent pairings impaired it. When the visual stimulus served as a prime, however, congruent priming at 500 ms and incongruent priming at 750 ms each reduced error rates in the most demanding auditory trials. This is a valuable demonstration of how crossmodal priming differs from multisensory integration.

Remaining in a naturalistic setting, Malzacher et al. varied levels of visual input—from free vision, to distorted vision via prism goggles, to full blindfolding—during an auditory-guided unrestricted navigation task. Their results show that the degradation of visual cues led to a strategic shift toward increased reliance on auditory cues, with frequency discrimination thresholds improving as a consequence. Reversing the direction of interaction, Chen et al. examined the potential role of auditory motion in influencing the perception of visual motion using a random dot kinematogram alongside task-irrelevant moving sounds. They reported auditory motion biasing decisions toward the heard direction but leaving the underlying visual motion sensitivity (thresholds) unchanged. Taken together, these diverse audiovisual studies demonstrate that auditory and visual signals interact systematically, shaping both behavior and neural processing.

Turning to the crossmodal correspondences contributions, the audiovisual axis gives way to less charted crossmodal territory. Lembke examined how untrained listeners identify gestures implied by environmental sounds through crossmodal matching with analogous visualizations. Gesture identification was reliable and largely independent of listeners' ability to recognize the underlying source material or causal action, and was barely improved by artificially emphasizing the gesture-relevant acoustic feature. Reymore and Lindsey investigated crossmodal correspondences between musical timbre and color (i.e., hue, saturation, and lightness) by asking listeners to match major scales, presented across different instruments and pitch registers, to colors drawn from a rigorously defined palette. The resulting associations were systematic, although complex and highly specific, and were influenced by both instrument type and pitch. Notably, ratings on semantic scales for timbre—which were also collected—successfully predicted listeners' color selections, particularly with respect to lightness and saturation.

This finding bridges naturally to the contribution by Zacharakis on the less explored auditory-olfactory modality pair. By asking participants to rate a set of timbres and scents along 12 shared descriptive scales, he demonstrated a common semantic organization spanning the two modalities. Perhaps more importantly, semantic distances between stimuli accounted for a substantial portion of the variance in previously estimated direct crossmodal distances, suggesting that shared meaning may constitute a key mechanism underlying auditory-olfactory correspondences. Pushing further into aesthetic territory, Antović et al. examined written corpora on Beethoven's first 10 piano sonatas in search of correspondences between linguistically expressed image-schemata (namely, Force, Path, Link, Balance, and Containment) and musical structures evident from the scores themselves. The major finding was that image-schematic structures in the music and in the language written about it corresponded systematically, both in their relative strength and in their directional tendencies.

The multisensory integration branch of this Research Topic advanced the field by examining a range of audiovisual scenarios using behavioral and neurophysiological methods, often in combination. By contrast, the crossmodal correspondences branch was smaller, yet conceptually broader, systematically addressing associations between sonic structures and colors, scents, gestures, and linguistically expressed image schemata. An important area not represented in the current Research Topic concerns associations and interactions between audition and touch or gustation, modality pairs that have shown considerable promise in both theoretical and applied research (Merchel and Altinsoy, 2020; Guedes et al., 2023; Rodríguez et al., 2023). A deeper understanding of crossmodal correspondences and multisensory perception carries broad applied potential—from the design of more naturalistic audiovisual environments and assistive technologies to the enhancement of creative and artistic practice, among other avenues exploiting the systematic ways in which the senses inform one another.
